# A Case of Recurrent Bacterial Meningitis Due to Retained 54-Year-Old Ureterodural Anastomosis

**DOI:** 10.1177/2324709618795293

**Published:** 2018-08-21

**Authors:** Carlos D’Assumpcao, Ahana Sandhu, Arash Heidari, Arman G. Froush, Shahab Hillyer, Joseph Chen, Alan Ragland

**Affiliations:** 1 Ross University, Miramar, FL, USA; 2 Kern Medical—University of California Los Angeles, Bakersfield, CA, USA

**Keywords:** arachnoid-ureterostomy, subarachnoid-ureteral shunt, uretero-arachnoid anastomosis, lumbar ureteral shunt, lumboureteral shunt, ureterodural anastomosis, recurrent meningitis, congenital hydrocephalus, hydrocephalus, ventriculomegaly, increased intracranial pressure, urinary tract infection, urinary retention, computed tomography myelogram

## Abstract

Ventriculoperitoneal shunts are the current treatment of choice for congenital hydrocephalus. It is rare for physicians to see patients with alternative types of shunting devices. Lumboureteral shunts, once popular from the 1940s to 1960s, decompress via the genitourinary system. Immediate complications were dehydration, electrolyte imbalances, infection, and the sacrifice of a functional kidney. Long-term complications include retrograde meningitis due to urinary tract infections. Three shunt types have been documented: polyethylene, silicone rubber, and ureterodural anastomosis. Routine imaging cannot detect a ureterodural anastomosis, and if suspected, computed tomography myelogram is needed for confirmation. This article presents the case of a man with long-standing ureterodural anastomosis that required ligation after recurrent episodes of acute meningitis secondary to urinary retention.

## Introduction

Modern ventriculoperitoneal (VP) shunts are currently the treatment of choice for hydrocephalus. The physician may rarely see patients with alternative types of shunting devices. Lumboureteral shunts were popular from the 1940s to 1960s. The ureterodural anastomosis described by Heile^1^ in 1925 was the first lumboureteral shunt. The primary advantage of this shunt type was the hydrodynamic resistance in the ureter was enough to preclude the need for a 1-way valve.^2^ Dr Donald Darrow Matson is credited with popularizing lumboureteral shunts^3-7^ by adapting the ureterodural anastomosis with the introduction of a polyethylene shunt to connect the ureter to the dural sac through the psoas muscle.^3^ Dr Matson felt the use of a tube made the procedure easier to complete with fewer complications, as he sought to improve adoption of the procedure.^4^ Immediate complications were dehydration, electrolyte imbalances, infection, and the sacrifice of a functioning kidney.^7^ Long-term complications include syringomyelia^8^ and retrograde meningitis^9^ due to urinary tract infections.^5,10^ Three documented shunt types have been used to decompress the cerebrospinal system via the genitourinary system: valveless polyethylene catheter,^3,8,11^ silicone rubber catheter,^12,13^ and a ureterodural anastomosis.^1,14,15^ Of the 3 types, ureterodural anastomosis may be invisible on routine imaging techniques. Presented here is a functional ureterodural anastomosis in a patient with recurrent acute meningitis.

## Case Presentation

A 59-year-old man, a carpenter by trade, with congenital hydrocephalus with reportedly multiple shunt revisions at a young age and a right nephrectomy at the age of 5 years was transferred from an outside hospital for a higher level of care. Two months prior, he underwent left hip arthroplasty and was discharged to a rehabilitation facility.

**Figure 1.**
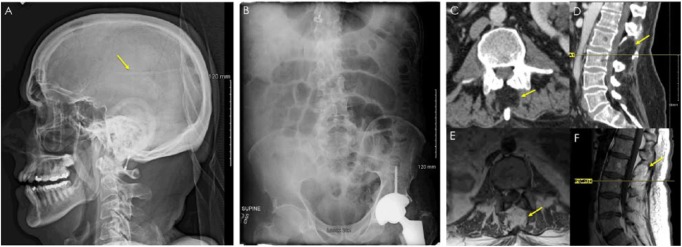
Initial imaging investigations. Proximal remnant of a ventricular atrial shunt is visible on lateral head view of X-ray shuntogram (A). No radiopaque abdominal shunt can be found on abdominal X-ray (B), although the left hip arthroplasty is visible. Computed tomography with intravenous contrast reveals an unknown lumbar process (C, D). Magnetic resonance imaging without contrast enhancement reveals the lumbar defect has the same T1 weighted signal as adipose tissue (E, F), suggesting possible postoperative changes of unknown chronicity.

During his stay at the rehabilitation facility, the patient had episodes of urinary retention, requiring a Foley catheter. He subsequently suffered a seizure, developing sixth nerve palsy, and nuchal rigidity. He was admitted to an outside hospital. It was reported that he had a *Pseudomonas aeruginosa* urinary tract infection and recurrent culture-negative meningitis. Imaging reported the presence of the proximal remnant of a ventricular shunt (Figure 1) that was placed in childhood. The patient received multiple courses of antibiotics including azithromycin, vancomycin, ceftriaxone, cefepime, and then meropenem. His course was complicated with the development of an allergic reaction to antibiotics and a pulmonary embolism.

On transfer to our institution, all antibiotics were stopped. The patient failed a trial of voiding with acute urinary retention. A Foley catheter was placed. The following morning, the patient developed a headache, sixth nerve palsy, and nuchal rigidity. A computed tomography (CT) scan of the head revealed worsening hydrocephalus. He was admitted to the intensive care unit and an external ventricular device was placed. Cerebrospinal fluid (CSF) studies revealed acute bacterial meningitis with glucose <1 mg/mL and grew *P aeruginosa* (aztreonam MIC [minimum inhibitory concentration] 1.5 µg/mL). Urine cultured extended spectrum β-lactamase *Klebsiella pneumonia* (aztreonam MIC >256 µg/mL) and *P aeruginosa* (aztreonam MIC 1.5 µg/mL; speciation and sensitivities by VITEK2 and ETEST, bioMérieux USA, Durham, NC) Based on these results, the patient was started on aztreonam and fosfomycin.

**Figure 2.**
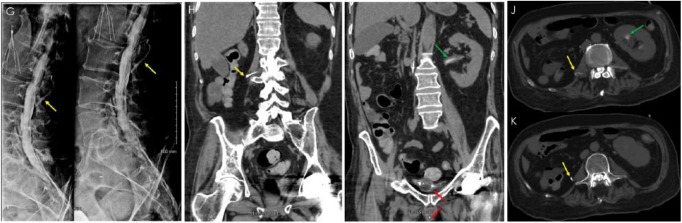
Computed tomography (CT) myelogram. X-ray myelogram with intrathecal iohexol contrast (Omnipaque, GE Healthcare, Buckinghamshire, UK) by fluoroscopy-guided sacral hiatus puncture (G) followed immediately by CT abdomen and pelvis without intravenous contrast (H, I, J, and K). Contrast is visible within an extra-thecal tubular structure beginning at approximately L2-L3 vertebrae (G, H, J, and K—yellow arrows). Contrast is also seen within the bladder surrounding the Foley catheter bulb and within the Foley catheter (I—red arrows), suggesting patent communication between the intrathecal space and the genitourinary system. Contrast in left renal pelvis suggests retrograde flow in the contralateral ureter (I and J—green arrows).

The family reported the presence of a “kidney shunt” placed in childhood. After a review of relevant medical literature, we suspected the presence of a ureterodural anastomosis as all imaging did not show evidence of functional hardware. After the resolution of meningitis, a CT myelogram was performed and revealed the presence of a patent ureterodural anastomosis (Figure 2).

**Figure 3.**
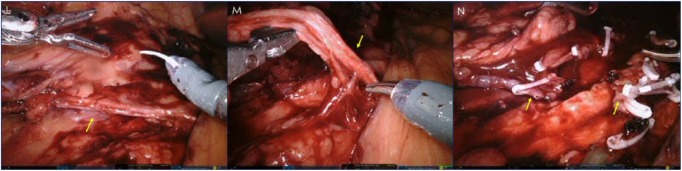
Robot-assisted laparoscopic exploration of right retroperitoneum: Left side of each frame is caudal. Right side of each frame is cephalic. Right ureter was identified (L), found to be well vascularized without acute signs of inflammation. Proximal end of abdominal segment of right ureter was isolated by blunt dissection (M) and associated collateral vascularization were identified and subsequently ligated. After the right testicular artery and vein as well as the right genitofemoral nerve were identified (not shown), the right ureter was ligated at its most proximally accessible end in the abdomen (N) without visible evidence of foreign shunt material.

The patient underwent the removal of the retained proximal remnant of ventricular shunt (*P aeruginosa*, 14 colony forming units [CFU] by semiquantitative culture of catheter tip). Further history obtained from the family revealed this ventricular shunt was the remnant of a ventricular atrial shunt that failed due to infection and required the removal of the distal end of the catheter. After completion of a 25-day course of aztreonam and confirmation of CSF sterility, a robot-assisted laparoscopic exploration of right retroperitoneum with ligation of right ureter was performed (Figure 3) immediately followed by a VP shunt placement during same operative session.

The patient was successfully discharged to an acute rehabilitation facility and subsequently home without recurrence of meningitis.

## Discussion

Congenital hydrocephalus is largely an obstructive hydrocephalus, most commonly caused by stenosis of the aqueduct of Sylvius.^16^ Untreated, the condition is devastating to the child. Historically, neurosurgical treatment options addressed CSF production, relief of obstruction, or diverting extra fluid.^2^ The VP shunt was first attempted in 1905 by German neurosurgeon Kausch.^17^ It fell out of favor due to frequent peritoneal occlusions.^2^ Other treatments included choroid plexectomy,^18^ third ventriculostomy,^19^ ureterodural anastomosis,^1,14,15^ endoscopic ventriculostomy,^20^ ventriculocisternostomy,^21^ cauterization of the choroid plexus,^22^ lumboureteral shunt,^3^ and competent ball valve on a ventriculoatrial shunt.^23^ Ventriculoatrial shunts suffered from frequent infections.^24^ The development of silicon rubber tubing addressed peritoneal occlusions and tissue reactions.^25^ One-way valves were improved on with the Holter valve^26^ and the anti-syphon device.^27^ With these improvements and the lack of better alternative treatments, VP shunts returned to favor after the 1960s,^28^ becoming the standard of care in congenital hydrocephalus.

Our medical literature review at the time of admission found that only ureterodural anastomosis and lumboureteral shunt fit the family’s description of a “kidney shunt.” Ureterodural anastomosis as described by Heile^1^ involved the sacrifice of a functional kidney, L2-L3 laminectomy, freed renal pelvis pulled through a psoas muscle tunnel to the laminectomy site, and anastomosed onto the dural sac. The ureter was chosen because it had the correct hydrodynamic resistance to retrograde flow in the form of ureteral peristalsis that precluded the need for a 1-way valve.^2^ Lumboureteral shunts were popularized by Dr Matson.^29^ Matson adapted Heile’s procedure by utilizing a polyethylene catheter to connect the ureter through a psoas muscle tunnel to the dural sac after a L2-L3 laminectomy.^3^ Later, silicone rubber catheter was also used as a conduit.^12^ Of these 3 shunt types, ureterodural anastomosis was the most likely to be radiolucent. This radiographic property contributed to the diagnostic challenge at initial presentation.

It should be mentioned that it is not known if radiopaque material were designed into shunts used in the 1940s to 1960s. Silicon rubber shunts have been reported to calcify and cause obstruction,^13^ possibly making them radiopaque in the process. Nonetheless, the authors’ prediction that the lumboureteral shunt was a ureterodural anastomosis due to its radiolucency and to the patient’s clinical presentation was ultimately proven correct.

In hindsight, there were radiographic clues of postsurgical changes. A lumbar process at the level of L2 vertebra was visible on initial CT. Magnetic resonance imaging corroborated this finding, suggesting postoperative changes of unknown chronicity (Figure 1). This fits with the reported description of the operative procedure where the spine and lamina of the second lumbar vertebra are removed.^1,3,14,15^ However, the authors found that this finding along with the patient’s history were not clinically specific enough to confirm the presence of the shunt without a CT myelogram.

Ultimately, the presence and patency of the lumboureteral shunt was confirmed by a CT myelogram with intrathecal iohexol contrast media (Omnipaque, GE Healthcare) administered by sacral hiatus puncture under fluoroscopic guidance (Figure 2). This modality was ideal as contrast in the dural sac could be followed as it drained. Retrograde pyelogram may push colonized urine into the dural sac and cause iatrogenic meningitis.^9^

Ligation of the shunt was necessary given the obstructive urinary symptoms associated with benign prostatic hyperplasia and neurogenic bladder. The resultant urinary retention with a patent ureteral connection to the subarachnoid space placed the patient at high risk of recurrent meningitis. Following its ligation, the patient’s communicating hydrocephalus was managed with a VP shunt, a viable replacement for the foreseeable future given the currently available medical experience in its management.

The 2017 Infectious Diseases Society of America Guidelines for Healthcare-Associated Ventriculitis and Meningitis^30^ do not address management considerations for patients with ancient thecal shunts of any type. The authors are not aware of any studies addressing the prevalence of ancient neurological shunt techniques. A review of the literature suggests it may be more common than may be assumed. Dr Matson reported in 1953 that 20 of the 50 pediatric patients were alive at 2-year follow-up. According to one biography of Dr Matson in 1983, “some of the patients [with hydrocephalus] so treated [with a lumbar-ureteral shunt] are still being seen by his successors.”^29^

The retained proximal section of the ventricular atrial shunt was removed as it was nonfunctional and, in the setting of *P aeruginosa* meningitis, placed the patient at increased risk of biofilm formation within the meninges. Semiquantitative culture of the catheter tip produced 14 CFU; however, there are no current established guidelines for semiquantitative studies of catheter tip in catheter-associated meningitis. One may assume that a sterile catheter tip with no CFU should be the goal.

Variations of lumboureteral shunts include operations without nephrectomy and ventriculoureteral shunts with or without nephrectomy. Ventriculoureteral shunt without a nephrectomy have been used successfully without reported complications of meningitis or dehydration.^31^ They even have been successfully revised.^32^ In 1993, Irby et al^33^ documented the long-term follow-up of 4 patients with ventriculoureteral shunts. All the 4 patients eventually required reoperation, albeit at a lower reoperation frequency than with standard shunts. It was recommended that ventriculoureteral shunts are used only in selected patients with multiple failures of standard shunts.^33^ Ventriculoureteral shunts have been used as recently as 2013 by Sarkar and Karthikeyan^34^ in India in a 60-year-old patient with tuberculosis meningitis and hydrocephalus.

## Conclusions

This case report describes the rediscovery of a ureterodural anastomosis, 54 years after placement. It was associated with recurrent retrograde severe bacterial meningitis after episodes of urinary retention. Its radiolucency contributed to the diagnostic challenge. If suspected, it may be confirmed with a CT myelogram. Surviving patients who received these shunts as children in the 1940s to 1960s may develop urinary retention from benign prostatic hypertrophy as they age and may present as this patient did. Physicians should be aware of the existence of alternative shunt types and how to confirm their presence if suspected. Future studies should address the prevalence of alternative shunt types.
